# Deep Learning for Brain MRI Artifact Correction: Current Challenges and Future Directions

**DOI:** 10.3390/bioengineering13060699

**Published:** 2026-06-18

**Authors:** Jiangfan Yu, Sibusiso Mdletshe, Hamid Abbasi, Eryn Kwon, Samantha Holdsworth, Alan Wang

**Affiliations:** 1 Department of Anatomy and Medical Imaging, Faculty of Medical and Health Sciences, University of Auckland, Auckland 1023, New Zealand; jiangfan.yu@auckland.ac.nz (J.Y.); sibusiso.mdletshe@auckland.ac.nz (S.M.);; 2 Auckland Bioengineering Institute, University of Auckland, Auckland 1010, New Zealand; h.abbasi@auckland.ac.nz; 3 Centre for Brain Research, Faculty of Medical and Health Sciences, University of Auckland, Auckland 1023, New Zealand; 4 Mātai Medical Research Institute, Gisborne 4010, New Zealand; 5 Medical Imaging Research Center, Faculty of Medical and Health Sciences, University of Auckland, Auckland 1023, New Zealand; 6 Centre for Co-Created Ageing Research, Faculty of Medical and Health Sciences, University of Auckland, Auckland 1023, New Zealand

**Keywords:** deep learning, brain MRI, artifact correction

## Abstract

Structural magnetic resonance imaging (sMRI) is progressively used to diagnose brain diseases; however, brain sMRI scans can be easily corrupted by artifacts, e.g., motion artifacts. To remove artifacts, deep learning (DL) algorithms have been extensively studied recently. However, their performance and the challenges currently faced in clinical practice (e.g., real-world robustness, hallucination and over-smoothing) have not been adequately studied in a quantitative manner. In this structured literature review, we quantitatively examined DL-based artifact correction studies (*N* = 30), retrieved from the major databases (i.e., Google Scholar, PubMed, Web of Science, and Scopus), which particularly focused on clinical-field-strength (defined as 1.5 Tesla (T) and above) sMRI in a non-pediatric setting. Our review suggests that current DL-based approaches exhibit promising fidelity measured by structural similarity (SSIM, 0.92 ± 0.05) index and peak signal-to-noise ratio (PSNR, 32.85 ± 4.53 dB). In addition, We identified the factors underlying hallucination or over-smoothing, which are associated with neural network (NN) architecture and the training process. This study also reveals the potential advantages, brought about by frequency-aware NN. Finally, we outline several future directions, including an emerging paradigm in DL, namely physics-informed NN (PINN).

## 1. Introduction

Structural magnetic resonance imaging (sMRI), e.g., T1-weighted (T1w), T2-weighted (T2w), Fluid-Attenuated Inversion Recovery (FLAIR) and cine magnetic resonance imaging (MRI), is increasingly applied to diagnose brain disease [1,2,3]. The diagnostic accuracy heavily relies on the image quality of sMRI scans, which can be particularly compromised by artifacts [4]. However, artifact correction for brain sMRI remains a challenge in post-processing (also referred to as retrospective correction) [4,5,6,7]. To cope with this challenge, deep learning (DL) algorithms have been intensively studied recently, showing promising results [4,6,7]. Given the growing number of DL-based artifact correction approaches [7], it is necessary to quantitatively assess their performance. Additionally, two aspects of the performance are crucial in clinical practice, which are fidelity (referring to the ability of preserving non-artifact-related information, e.g., anatomical structures, signal intensity and image clarity) and the robustness against the domain gap between synthetic and real-world data (also known as real-world robustness) [8,9].

### Related Work

A few studies [4,5,6,7,10] have qualitatively examined studies that were not confined to brain sMRI between 2009 and 2024 [4,5,6,7,10]. A.S. Lundervold et al. [5] examined previous studies up to the year 2018, offering the conceptual explanations for four major neural network (NN) architectures, i.e., vanilla U-net, densely connected network (DenseNet), generative adversarial network (GAN) and residual network (ResNet). Until 2020, DL-based approaches were rarely explored to remove volumetric (also known as out-of-plane) artifacts, as stated by S. Lee et al. [10]. From 2020 onwards, a large portion of works were built upon the above NN architectures [4,6,7], i.e., U-net [11,12,13,14,15,16,17,18,19]; DenseNet [20,21]; GAN [21,22,23]. Meanwhile, newly developed NNs, including deep convolutional NN (CNN) [9,24], auto-encoder [25,26,27], Cycle-Consistent Generative Adversarial Network [28,29,30] and Vision Transformer (ViT) [31], were also introduced to this area. Among these, Cycle-Consistent Generative Adversarial Network extended the standard GAN design by incorporating bidirectional mapping-based data augmentation, which helps to bridge the gap between two image domains [32]. ViTs could transform MRI images into a sequence of learnable embeddings through patch-based tokenization [31].

During the period 2018–2023, among different types of artifacts (e.g., intensity inhomogeneity [18,29], ghosting [33] and geometric distortion [34]), motion artifacts, defined as image degradation attributed to involuntary movement during MRI scanning, drew significant attention, as implied by V Spieker et al. and S. Lee et al. [6,10]. In their work, these motion artifacts were categorized by the cause, which is either (1) rigid motion, e.g., head rotation, or (2) non-rigid (elastic) motion, typically referring to tissue deformations. According to R Singh et al. [7], several DL-based approaches were developed to address rigid motion artifacts. In contrast, elastic motion artifact correction using DL algorithms was barely available [6,10].

On the other hand, in recent years (i.e., between 2020 and 2025), the frequency-aware NNs [20,34] were preliminarily studied to integrate k-space information, i.e., the frequency-domain (spectral) representation of sMRI scans. The adaptive attention mechanism (AAM) was incorporated into NNs [18,21,25,31,35], which supports selective correction by weighting regions with pronounced artifacts. Moreover, an emerging paradigm that uses physics-based regulation, the physics-informed NN (PINN), was introduced recently in DL [34,36]. In addition to advanced NN architectures, innovative learning strategies have also been explored to address the heavy dependence of NNs on large amounts of labeled, paired (i.e., artifact-corrupted and artifact-free (clean) scans acquired from the same individual) clinical data. These strategies include unpaired learning [28,29,30] and unsupervised learning [19,26,31,34]. In terms of unsupervised learning, deep image prior (DIP) [26,34] is a typical example, as it could learn non-artifact-related information directly from the corrupted scan, without requiring external training data.

In contrast to the above advancements, the performances of current artifact correction approaches have rarely been reviewed in a quantitative manner. Currently, there is no review specifically focusing on brain sMRI, although it brought more challenges in achieving high-fidelity correction [4,6,7]. In this study, we aimed to assess DL-based retrospective correction approaches for brain sMRI, and critically evaluate their fidelity and the clinical robustness. Additionally, the present study is the first of its kind to examine PINN-based studies.

## 2. Materials and Methods

**Figure 1:**
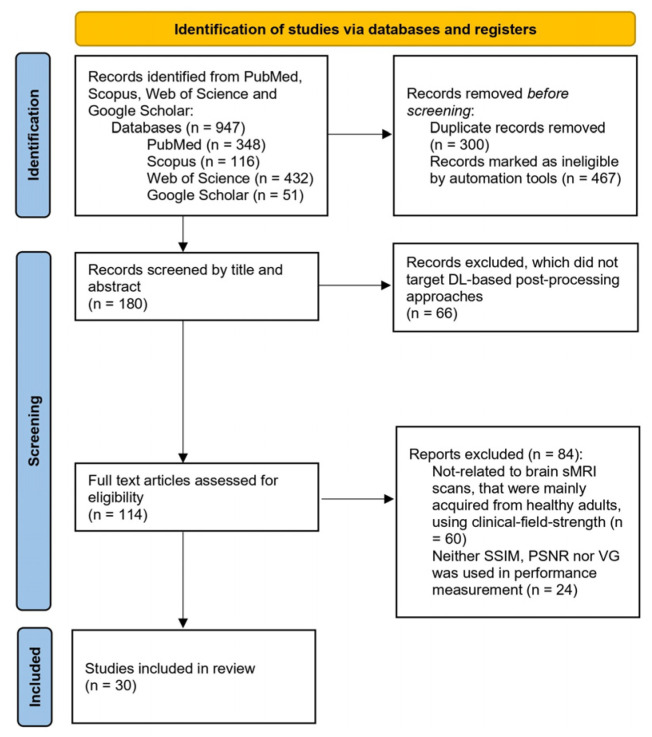
PRISMA diagram.

Following the Preferred Reporting Items for Systematic Reviews and Meta-Analyses (PRISMA) guidelines [37], we used the structured methodology (Figure 1) to retrieve the literature, including journal articles, conference papers and preprints, whose publication dates range from 1st January 2020 to 31st December 2025. It is important to note that DL-based denoisers typically can be applied to remove artifacts [38]. Consistent with prior reviews [5,6], DL-based studies targeting denoising or artifact correction for brain sMRI were searched across four academic databases (i.e., Google Scholar, PubMed, Web of Science, and Scopus) over 2 weeks (i.e., from 1st to 15th February 2026) using queries (see Supplementary Table S1), e.g., the PubMed query used was

(“magnetic resonance imaging”[tiab] OR MRI[tiab]) AND (artifact*[tiab] OR artefact*[tiab] OR noise*[tiab]) AND (correct*[tiab] OR remov*[tiab] OR reduc*[tiab] OR denois*[tiab]) AND (“deep learning”[tiab] OR “neural network*”[tiab]) AND (brain[tiab]) AND (“2020/01/01“[dp]:”2025/12/31”[dp])

The linguistic variations were incorporated into the above search queries, including alternative spellings (e.g., artifact vs. artefact), singular and plural forms (e.g., artifact*, in which the wildcard, denoted by *, stands in for one or more unknown characters, therefore allowing artifacts), and abbreviation/full-form pairs (e.g., Magnetic Resonance Imaging vs. MRI).

To improve efficiency, we built a Python (version 3.8.18) script, using PyCharm 2024.1 (Community Edition), to filter out records most likely targeting non-brain sMRI, low-field, the presence of brain pathology and other irrelevant research by searching terms (see Table 1) in the title/abstract before manual screening. These terms were carefully selected to minimize the risk of the Python script excluding eligible studies. To further address the potentially missed eligible studies, we manually cross-checked the studies included in this study against those examined by previous reviews [4,6,7,10]. The absence of falsely excluded records in this cross-checking exercise suggests that our automated Python-based filtering would not introduce a substantial bias.

During screening, the title, abstract, full-text, and the de-duplicated entries (also referred to as records) were accepted when they met the following inclusion criteria:

• The testing data included at least one imaging modality of sMRI.
• The brain sMRI scans were majorly acquired from healthy subjects, with no less than 1.5 T magnetic field strength.
• The DL-based approaches aimed for neither fetal nor infant MRI.
• Used the consistent performance evaluation metric, i.e., either structural similarity index (SSIM), peak SNR (PSNR) or visual grading (VG) by the radiologist.

Notably, to comply with the above 2nd criterion, studies targeting scans for patients with known or suspected brain-disease were excluded in the present review. The main reason for this exclusion is that disease-related features were likely to confound NNs, which were designed to correct healthy subjects’ sMRI data. As evidenced by M. Rotman et al.’s [39] work, the performance (i.e., evaluated by normalized mean square error (NMSE)) of DL-based approaches might vary substantially across brain disease (NMSE: 0.043) and healthy (NMSE: 0.012) datasets. Additionally, we excluded works focusing on MRI with <1.5 T magnetic field strength (also referred to as low-field) to suppress the biases associated with field strength on scans’ image quality, particularly measured by the signal-to-noise ratio (SNR). As stated by T. Tajima et al. [24] and T. Ssentamu et al. [40], the higher the magnetic field strength in MRI, the better the overall image quality (e.g., 4.17 SNR was a result of low field sMRI, where clinical-field-strength sMRI led to 21 SNR). Moreover, according to T.K. Turesky et al. [41] and J. Chin et al. [42], DL-based methods developed for brain sMRI scans of adults were hardly applicable to that of fetal or infant (defined as younger than 5 years old) children. It is probably due to their smaller brain size, compared with adults, resulting in a decrease in contrast in sMRI images. Hence, studies targeting scans collected from fetuses or infants were not included in this review. An additional reason supporting the above exclusion is that fetal MRI [42], low-field MRI [43] and brain-disease patient MRI [44,45] have been addressed separately in previously published DL-focused systematic reviews. Therefore, clinical-field-strength brain sMRI of non-pediatric healthy subjects was targeted as a rational scope for the present review. This narrowed scope possibly decreases the heterogeneity of the included studies, permitting the subsequent quantitative comparison.

At the eligibility assessment stage, to implement quantitative comparison afterwards among DL-based post-processing approaches, studies were regarded as eligible, only if they used the consistent performance evaluation metric or VG by the radiologist. More specifically, SSIM and PSNR have been applied with the highest frequency to evaluate the fidelity in this context [4,6,7,10]. By comparing a pair of corrected and clean scans, SSIM and PSNR separately measure how much the anatomical structures and signal intensity are consistent between them [4,6,7,10]. Also, it is important to note that clean scans (ground truth) might not be available for in vivo validation. Usually, in such cases, VG implemented before and after correction were analyzed with Cohen’s kappa or the Wilcoxon signed-rank test to assess whether there was perceptual image quality improvement [8].

To facilitate quantitative comparison across included studies, we extracted key details, e.g., SSIM and PSNR-based validation results, from each of them. Moreover, following Y. Zhao et al.’s work [25], the conversion rate, i.e., one 3D sMRI scan = 600 sampled 2D images (also referred to as slices), was adopted to standardize the reported training data volumes. To evaluate the relationship between PSNR/SSIM scores and training data size or resolution, the Pearson correlation coefficient (denoted by *r*, presented in Equation (1)) was applied, followed by the probability (*p*) value calculation using a t-distribution with *N*-2 degrees:

(1) $$r=\frac{\sum \left(x_{i}-\bar{x})(y_{i}-\bar{y}\right)}{\sqrt{\sum {\left(x_{i}-\bar{x}\right)}^{2}{\left(y_{i}-\bar{y}\right)}^{2}}}$$

where x_i_ represents the training data size or resolution of study i and y_i_ denotes the metric score reported by study i.

## 3. Results

The important details for each reviewed study are listed in Table 2 (more information, including training data volume and input data size, is provided in Supplementary Table S2) and are associated with input data, NN architectures and learning strategies. In the characteristics column, frequency-aware and 3D-aware, respectively, denote the NN that dealt with spectral and volumetric data. As for the Validation Dataset Property column, the meaning of both is that the proposed approach was tested with synthesized and real-world (denoted as in vivo) scans simultaneously. Additionally, the obstacle column summarizes whether typical fidelity-related obstacles, including hallucination (referring to spurious or distorted anatomical structures), over-smoothing (i.e., manifesting as pixelated edges or blurred details, corresponding to degraded image clarity), and poor real-world robustness, were encountered in the reviewed studies.

From the data in Table 2, it is apparent that using DL to remove in-plane (i.e., confined in the slice) artifacts, particularly that result from rigid motion, was extensively explored. More specifically, just over one-third of the studies (*N* = 11) were found to target rigid motion artifacts, whereas non-rigid motion artifact correction was rarely researched, except by G.G. Potter et al. [13]. About four-fifths of the reviewed studies (*N* = 24) aimed at restoring corrupted brain MRI slices, and most of them (*N* = 20) were based in the spatial domain.

An important finding is that, surprisingly, there were no frequency-aware NNs that can address volumetric artifacts for brain sMRI scans. A possible explanation for this might be insufficient research on how to integrate spectral representations of sMRI scans into NNs, as suggested by only a small proportion (i.e., 4 out of 30) of studies using frequency-aware NNs. Moreover, to remove out-of-plane artifacts, six NNs were developed to receive 3D spatial-domain-based input data, including the three-slice stacked format.

It is expected that 86.67% (*N* = 26) of the studies took the above obstacles (see Table 2) into account. In particular, the challenges of hallucination (*N* = 12) and over-smoothing (*N* = 12) were more prevalent than low real-world robustness (*N* = 6) among these studies.

Caution needs to be applied when interpreting PINN-related data due to the limited studies available [34,35,36]. Although it is notable that the peak scores of SSIM (0.99) and PSNR (41.35 dB) were achieved by the PINN-based study [34], this finding remains preliminary.

Additionally, to enhance real-world robustness, innovative learning strategies, e.g., unpaired learning, residual learning and unsupervised learning, were implemented in approximately 40% (*N* = 12) of included studies. The present review found that there were no significant discrepancies between the conventional fully supervised DL (averagely PSNR = 32.83 ± 4.76 dB, SSIM = 0.93 ± 0.06) and those trained with innovative strategies (averagely PSNR = 32.62 ± 4.31 dB, SSIM = 0.91 ± 0.05) in terms of PSNR, SSIM-measured performance. Moreover, no strong correlations (defined as *p* < 0.05 [51]) were revealed between PSNR, SSIM scores and training data size or sMRI scans’ resolution (see Table 3) in the present study.

## 4. Discussion

Our aim in this review was to quantitatively assess the applications of DL in correcting artifacts for brain sMRI in non-pediatric healthy subjects. We examined the metrics (i.e., SSIM, PSNR)-based validation results among the included DL-based approaches, and analyzed their real-world robustness as well as fidelity.

### 4.1. Real-World Robustness Obstacle

Table 4 compares SSIM and PSNR scores of studies, whose validation datasets were categorized as Both (see Table 2). It appeared that overcoming real-world robustness was still challenging, as suggested by the overall decrease in PSNR, shown in in vivo validation, compared to that coming from synthesized datasets. Notably, the above trend must be interpreted with caution due to the limited data available and warrants further investigation on a larger scale. It is unexpected to observe that M. Kidoh et al. [9] and B.A. Duffy et al. [15] achieved in vivo SSIM results that were higher than synthesized data-based ones. The above outlier scores may reflect the curation of clean ground truths. More specifically, in the curation process of the above studies [9,15], these brain sMRI scans were treated as clean and were in fact acquired using doubled field strength (i.e., 3 T, whereas 1.5 T-based scans were regarded as artifact-corrupted) [15] or the increasing number of acquisitions (NAQ = 5, whereas NAQ = 2 meant that the corresponding scans contained artifacts) [9]. On the contrary, we found no strong evidence that brain sMRI with either 3 T field strength or 5 NAQ is immune to artifacts. Thus, the above curated ground truths might not be completely free from artifacts, which could contribute to the elevated SSIM scores.

### 4.2. Quantitative Fidelity Evaluation

**Figure 2:**
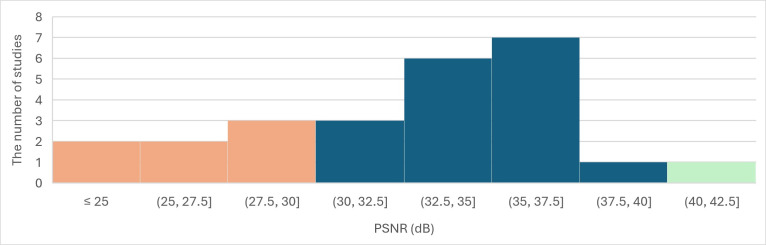
PSNR scores’ distribution across included studies.

**Figure 3:**
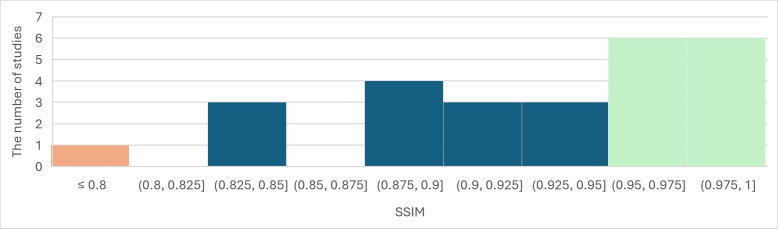
SSIM scores’ distribution across included studies.

Our study suggested that, from 2020 to 2025, DL-based approaches exhibited promising results in restoring brain sMRI scans. Overall, the image quality, particularly fidelity quantified by SSIM and PSNR, shall be regarded as acceptable, as the majority of reviewed approaches passed the cut-off thresholds (i.e., SSIM > 0.8, PSNR > 30 dB) [52,53] (see Figure 2 and Figure 3).

However, the suboptimal fidelity (defined as SSIM ≤ 0.95, PSNR ≤ 40 [52,53]) remained a challenge for DL-based approaches. Particularly, as presented in Figure 2, the largest number of studies were in 32.5–37.5 dB groups. Just over 50% of studies (*N* = 14) demonstrated less than 0.95 SSIM (see Figure 3 Consistent with V. Venkatesh et al. [18], such a challenge might be implied by the prevalence of hallucinations and over-smoothing that were observed in the Section 3.

The higher the PSNR, the lower the pixel-wise intensity differences, reflecting greater fidelity. Aligned with A. Senthil Anandhi et al. [52], ≤30 dB (highlighted in orange) indicates severe degradation, whereas >40 dB (highlighted in green) suggests an outstanding consistency.

Above all, SSIM ranges from 0 to 1. The higher SSIM score signifies that the more anatomical structures are maintained. More specifically, ≤0.8 (highlighted in orange) indicates visible distortion, in contrast to >0.95 (highlighted in green) implying a nearly intact structure [52,53].

Frequency-aware NNs were found to likely show relatively high PSNR performance (i.e., averagely 37.04 ± 2.99 dB), exhibiting a nearly 5 dB increase on average, compared to spatial-domain-based NNs (i.e., averagely 32.06 ± 4.55 dB). More specifically, among the peak PSNR groups (i.e., (37.5–42.5]), all studies [20,34] were using frequency-aware NNs. In agreement with M.S. Hosseini et al. [34], it seemed to be related to information redundancy in k-space, which is rarely present in the spatial domain.

Additionally, NNs embedded with AAM (denoted by adaptive attention in Table 2) appeared to play a facilitating role, in terms of SSIM. Overall, the anatomical structures were well-preserved (i.e., implied by SSIM ≥ 0.95) in studies [18,21,31,35] that integrated AAM. Aligned with the opinion of O. Dabrowski et al. [20], there was a risk that clean anatomical structures may vary after DL-based artifact correction, likely due to NNs restoring images in an unselective manner. Whereas artifacts are possibly regionally confined in brain sMRI scans [20,25]. To address this issue, AAM was introduced to promote region-wise correction, therefore potentially bringing benefits to SSIM performance.

### 4.3. Underlying Causes of Hallucinations

Hallucinations appeared to result from the insufficient fidelity of DL-based models. Aligned with V. Venkatesh et al.’s [18] points of view, a contributing factor to hallucinations was single-scale NNs (i.e., typically with one receptive field size), because they can hardly capture multi-scale features, e.g., the shape of brain structures, in sMRI scans, therefore leading to decreased fidelity in general. In contrast, multiple-scale NNs [18,25,27,31] showed few false anatomical structures.

Another potential cause of limited fidelity was likely due to DL-based approaches, particularly those based on GANs [28,29], having the chance to alter non-artifact-related information (also referring to as over-compensation). In agreement with works of J. Lee et al. [11], O. Dabrowski et al. [20] and L. Zhang et al. [12], we believe the hallucinations are associated with over-compensation. Accordingly, the preliminary findings (i.e., the peak SSIM/PSNR scores in the PINN subgroup) may be taken to suggest that physics-based regularization could help to suppress over-compensation, thereby presumably reducing the over-compensation-related hallucinations. It is necessary to emphasize that this interpretation remains inconclusive, because of the small number of available studies [34,35,36].

### 4.4. Underlying Causes of Over-Smoothing

Our review implies that over-smoothing, an effect that hinders DL-based models predicting high fidelity images, was probably related to the training process of NNs. It is important to note that the employed loss function (loss) plays a key role in this process [4,5,6,7,10]. Consistent with previous studies [18,21,26], L2 loss, i.e., mean square error (MSE), was prone to promote over-smoothing, given its poor sensitivity to detailed anatomical information. Whereas, L1 loss (i.e., Mean Absolute Error (MAE)) [18,21] and the hybrid losses, consisting of perceptual loss [12], adversarial loss [27,29] or SSIM loss [26], seemed to be advantageous to support NNs capturing this detailed information, as suggested by a clearer image, compared to that based on L2 loss [26].

Additionally, the occurrence of over-smoothing might be associated with severe artifact corruption, typically defined as over 30% of k-space data being distorted [15]. In this review, we found that overall, among studies experiencing over-smoothing (see Table 2), over-smoothing appeared to be more pronounced in studies involving severe artifacts. A plausible explanation was that NNs may face a trade-off between artifact removal and image clarity preservation and tend to blur anatomical details if poorly trained, aligning with A. Saini et al.’s [54] perspectives.

### 4.5. Study Limitations

We acknowledge several associated limitations for this review, since they might affect the generalizability, accuracy or robustness of the findings. Overall, it was non-trivial to balance the trade-off between narrowing the scope of the review and reducing the heterogeneity across the included studies. Due to the eligibility criteria (see Section 2), the findings from this review may not be generalized beyond clinical-field-strength brain sMRI of non-pediatric healthy subjects. Even within this narrowed scope, the heterogeneity arising from differing modalities, targeted artifacts and input data sizes remained among the included studies, likely decreasing the accuracy of the quantitative comparison. More specifically, we found no explicit reporting of meaningful sensitivity to sMRI modalities, implying that modality was unlikely to be the decisive element determining the DL’s fidelity in this context. Regarding DL’s transferability to different artifact types or varying input data size, there seemed to be no consensus, and further research is warranted. Although no strong correlation was found between SSIM, PSNR-measured fidelity and varying training size or resolution, the implication of which cannot be completely overlooked. Finally, in agreement with previous research [8,9], the suboptimal real-world robustness was treated as one of the current challenges in the present study; however, the number of available samples (*N* = 6) was restricted, therefore potentially limiting the robustness of the related interpretation.

### 4.6. Future Directions

The findings of our study have the potential to outline the future direction in this area. First, unsupervised or unpaired learning strategies show promise for enhancing real-world robustness by facilitating NNs learning from real-world datasets. Meanwhile, they exhibited comparable fidelity to that of fully supervised learning, with only a 0.2 dB decrease in PSNR and a 0.02 decrease in SSIM. Secondly, frequency-aware NNs were likely capable of enhancing PSNR performance by approximately 5 dB. In addition, 3D-aware NNs were expected to correct out-of-plane artifacts with acceptable fidelity (i.e., PSNR 35.21 ± 1.70 dB, SSIM 0.94 ± 0.08). Given the lack of NNs with both frequency and 3D awareness in this context, it seems to be an interesting topic for future research. Moreover, preliminary evidence suggests that PINN-based approaches may help reduce hallucination risk, but this conclusion remains limited by the small number of available studies.

## 5. Conclusions

This work thoroughly examined the current DL-based approaches for correcting artifacts in clinical-field-strength brain sMRI scans, acquired from non-pediatric healthy subjects. Specifically, it not only provided an assessment of fidelity in a quantitative manner, but also identified three common obstacles, i.e., suboptimal real-world robustness, hallucinations and over-smoothing, followed by presenting their potential causes. In this review, we revealed the quantified performance of the existing approaches, which can serve as the baseline for the related research. Additionally, the potential advantages that frequency-aware NNs, PINN and unsupervised learning strategies might bring about, were also stated, although they were newly introduced. A future research direction, i.e., frequency- and 3D-aware NNs, was suggested in this study, given that they were not currently available in this field.

## Figures and Tables

**Table 1:** Terms used in the Python script.

Reasons for Exclusion	Terms
Not related to the brain	cardiac, heart, liver, spine, abdomen, abdominal, knee, lung
Presence of brain pathology	tumour, tumor, cancer, glioma, glioblastoma, Parkinson, Alzheimer
In the pediatric setting	fetal, foetal, infant, childhood
Modalities other than sMRI	diffusion, CT
Below clinical-field-strength	low field
Not related to artifact correction	classification, detection, radiotherapy, dosage, EEG, harmonization, Super-resolution

**Table 2:** The details of the included studies.

Study	Data			NN				
	Datasets Accessibility	Validation Data Property	Resolution (mm^2^ or mm^3^)	Architectures	Characteristics	Target	Learning Strategy	Obstacles ^1^
M. Kidoh et al. [9]	Private	Both	0.39 × 0.39	Deep CNN		Noise	Fully Supervised	Not reported (NR ^3^)
J. Lee et al. [11]	Private	Synthesized	0.86 × 0.86	U-Net		(In-Plane, Rigid) Motion Artifact	Fully Supervised	Hallucination
L. Zhang et al. [31]	Private	Synthesized	1.0 × 1.0	Vit	Multi-scale Adaptive attention	(In-Plane, Rigid) Motion Artifact	Unsupervised Patch-wise	NR
O. Dabrowski et al. [20]	Private	Both	1.0 × 1.0	DenseNet	Frequency-aware	(In-Plane, Rigid) Motion Artifact	Fully Supervised	Hallucination
L. Zhang et al. [12]	Private	Synthesized	1.0 × 1.0	U-Net		(In-Plane, Rigid) Motion Artifact	Two-Stage Fully Supervised	Hallucination over-smoothing
G.G. Potter et al. [13]	Public	Synthesized	1.0 × 1.0	U-Net	3D-aware	(Out-Of-Plane, Elastic) Motion Artifact	Fully Supervised	Over-smoothing
Y. Zhao et al. [25]	Public	Synthesized	1.0 × 1.0	Auto-encoder	Multi-scale Adaptive attention	Ringing Artifact	Fully Supervised	Hallucination
T. Tajima et al. [24]	Private	In vivo	0.3 × 0.3	Deep CNN		Noise	Fully Supervised	Over-smoothing
I. Oksuz [14]	Public	Synthesized	1.0 × 1.0	U-Net		(In-Plane, Rigid) Motion Artifact	Fully Supervised	NR
B.A., Duffy et al. [15]	Public	Both	1.15 × 1.15 × 1.1	U-Net	3D-aware	(Out-Of-Plane, Rigid) Motion Artifact	Fully Supervised Patch-wise	Over-smoothing
S. Liu et al. [16]	Private	Synthesized	0.8 × 0.8	U-Net		Noise	Unpaired Learning	Low real-world robustness
S. Jung et al. [17]	Private	In vivo	0.95 × 0.95	U-Net		(In-Plane, Rigid) Motion Artifact	Unpaired Learning	Low real-world robustness
S. Li et al. [22]	Public	Both	1.0 × 1.0	GAN		Blurring Artifacts	Fully Supervised	Low real-world robustness
K. Pawar et al. [8]	Public	In vivo	0.94 × 0.94	Resnet		Noise	Fully Supervised	Over-smoothing
J. Liu et al. [46]	Private	Synthesized	0.75 × 1.05	Resnet		(In-Plane, Rigid) Motion Artifact	Residual Learning Patch-wise	Over-smoothing
S. Li et al. [47]	Public	Synthesized	1.0 × 1.0	Resnet		Rician Noise	Residual Learning Patch-wise	Over-smoothing
G. Chen et al. [28]	Public	Synthesized	0.75 × 0.75	Cycle-Consistent Generative Adversarial Network		(In-Plane, Rigid) Motion Artifact	Unpaired Learning Patch-wise	Hallucination Over-smoothing
T. Li et al. [29]	Public	Both	1.0 × 1.0	Cycle-Consistent Generative Adversarial Network		Intensity Inhomogeneity	Unpaired Learning	Hallucination
M.S. Hosseini et al. [34]	Public	In vivo	1.0 × 1.0	PINN	Frequency-aware	Geometric Distortion	Unsupervised	Low real-world robustness
G. Oh et al. [30]	Public	Synthesized	0.7 × 0.7 × 0.7	Cycle-Consistent Generative Adversarial Network	Frequency-aware	(In-Plane, Rigid) Motion Artifact	Unpaired Learning	NR
M. Moreno López et al. [19,48,49] ^2^	Public	Synthesized	NR	U-net	Frequency-aware	Gaussian Noise	Unsupervised	Over-smoothing
	Public	Synthesized	NR	U-Net		Gaussian Noise	Unsupervised	Over-smoothing
Y. Zhu et al. [26]	Public	Synthesized	NR	Auto-encoder		Gaussian Noise	Unsupervised	Over-smoothing
V. Venkatesh et al. [18]	Public	Synthetized	NR	U-net	Multi-scale Adaptive Attention	Intensity Inhomogeneity	Fully supervised	Hallucination over-smoothing
K. Pawar et al. [23]	Private	Both	1.0 × 1.0 × 1.0	Resnet		(In-Plane, Rigid) Motion Artifact	Fully supervised	Low real-world robustness
Y. Xu et al. [21]	Public	Synthetized	0.7 × 0.8	Residual DenseNet	3D-aware Adaptive attention	Rician Noise	Fully supervised	Over-smoothing
M. Ghaffari et al. [50]	Public	Synthetized	0.7× 0.7× 0.7	GAN	3D-aware	Blurring and Ringing Artifacts	Fully supervised Patch-wise	Hallucination
M. Ghahremani et al. [27]	Public	Synthetized	1.0 × 1.0	Auto-encoder	Multi-scale	Rician Noise Intensity Inhomogeneity	Fully supervised	Hallucination
M. Safari et al. [35]	Public	Both	1.0 × 1.0 × 1.0	PINN	Adaptive attention	(In-Plane, Rigid) Motion Artifact	Fully supervised	Hallucination Low real-world robustness
B. Nghiem et al. [33]	Public	Both	1.0 × 1.0 × 1.0	U-Net	3D-aware	Ghosting Artifact	Fully supervised	Hallucination
A. De Goyeneche Macaya et al. [36]	Public	Synthetized	1.0 × 1.0 × 1.0	PINN	3D-aware	Blurring Artifact	Fully supervised Patch-wise	Hallucination

^1^ We assigned hallucination and over-smoothing obstacles to the included studies if they explicitly regarded the above two obstacles as challenges, or if noticeable manifestations (e.g., distorted anatomical details) were observed in the reported corrected images [8,19,26,50] by J.Y. As for low real-world robustness, it was mostly inferred based on the reported SSIM and PSNR scores. ^2^ The evaluation study [19] tested two DL-based approaches, separately proposed by S. Soltanayev et al. [48] and S. Laine et al. [49]. Although these evaluated studies, i.e., Refs. [48,49] were published before 2020, their quantitative performance, reported after 2020, was included in the following analysis. ^3^ NR indicates that none of the above three obstacles were explicitly reported, observed by J.Y., or could be reasonably inferred.

**Table 3:** Pearson correlation coefficient with the 95% confidence interval (CI) and probability values.

	SSIM	PSNR
Training Data Size	*r* = 0.21 (95% CI −0.26 to 0.60) *p* = 0.37	*r* = 0.18 (95% CI −0.30 to 0.59) *p* = 0.46
Resolution	*r* = −0.42 (95% CI −0.74 to 0.06) *p* = 0.08	*r* = −0.48 (95% CI −0.78 to 0.00) *p* = 0.05

**Table 4:** Synthesized and in vivo performance validation.

Study	Validation Data Property	PSNR (dB)	SSIM
M. Kidoh et al. [9]	Synthesized	31.00	0.85
	In vivo	29.63	0.98
B.A., Duffy et al. [15]	Synthesized	35.00	0.98
	In vivo	33.34	0.99
T. Li et al. [29]	Synthesized	37.47	0.98
	In vivo	34.26	0.96
K. Pawar et al. [23]	Synthesized	NR ^1^	0.93
	In vivo	NR	0.91
M. Safari et al. [35]	Synthesized	36.01	0.97
	In vivo	32.37	0.85
B. Nghiem et al. [33]	Synthesized	NR	0.97
	In vivo	NR	0.88

^1^ NR denotes that the corresponding metric score was unavailable.

## Data Availability

No new data were created in this study, therefore data sharing is not applicable.

## Supplementary Materials

The following supporting information can be downloaded at: https://www.mdpi.com/article/10.3390/bioengineering13060699/s1, Table S1: Database-specific search strings; Table S2: The details of included studies.

## Abbreviations

The following abbreviations are used in this manuscript:

AAM	Adaptive Attention Mechanism
CNN	Convolutional Neural Network
DL	Deep Learning
FLAIR	Fluid-Attenuated Inversion Recovery
GAN	Generative Adversarial Network
MAE	Mean Absolute Error
MSE	Mean Square Error
NAQ	Number of Acquisitions
NMSE	Normalized Mean Square Error
NN	Neural Network
NR	Not Reported
PINN	Physics-Informed Neural Networks
PRISMA	Preferred Reporting Items for Systematic Reviews and Meta-Analyses
PSNR	Peak Signal-to-Noise Ratio
ResNet	Residual Network
sMRI	Structural Magnetic Resonance Imaging
SNR	Signal-to-Noise Ratio
SSIM	Structural Similarity Index
VG	Visual Grading
ViT	Vision Transformer
