# Supplementary material for: Deep Learning for Brain MRI Artifact Correction: Current Challenges and Future Directions
Source: Bioengineering (Basel). 2026 Jun 18;13(6):699. doi: 10.3390/bioengineering13060699 (PMC13295345; doi:10.3390/bioengineering13060699)
Supplement: Supplementary file 1 [file bioengineering-13-00699-s001.zip › bioengineering-4374252-supplementary.pdf]

**Table S1.** database-specific search strings

| Database | Query String * |
|---|---|
| PubMed | ("magnetic resonance imaging"[tiab] OR MRI[tiab]) AND (artifact*[tiab] OR artefact*[tiab] OR noise*[tiab]) AND (correct*[tiab] OR remov*[tiab] OR reduc*[tiab] OR denois*[tiab]) AND ("deep learning"[tiab] OR "neural network*"[tiab]) AND (brain[tiab]) AND ("2020/01/01"[dp] : "2025/12/31"[dp]) |
| Scopus[1] | TITLE-ABS-KEY ( ( "magnetic resonance imaging" OR MRI ) AND ( artifact* OR artefact* OR noise* ) AND ( correct* OR remov* OR reduc* OR denois* ) AND ( "deep learning" OR "neural network*" ) AND ( brain ) AND (retrospective OR post-process* ) ) AND PUBYEAR > 2019 AND PUBYEAR < 2026 |
| Web of Science | (TI=(("magnetic resonance imaging" OR MRI) AND<br>(artifact* OR artefact* OR noise*) AND<br>(correct* OR remov* OR reduc* OR denois*) AND<br>("deep learning" OR "neural network*") AND<br>(brain))<br>OR<br>AB=(("magnetic resonance imaging" OR MRI) AND<br>(artifact* OR artefact* OR noise*) AND<br>(correct* OR remov* OR reduc* OR denois*) AND<br>("deep learning" OR "neural network*") AND<br>(brain)))<br>AND PY=(2020-2025) |
| Google scholar[2] | (intitle:"magnetic resonance imaging" OR intitle:MRI) AND (intitle:artifact OR intitle:artifacts OR intitle:artefact OR intitle:artefacts OR intitle:noise OR intitle:noises) AND (intitle:correction OR intitle:correct OR intitle:remove OR intitle:removal OR intitle:reduce OR intitle:reduction OR intitle:denoising OR intitle:denoise) AND (intitle:"deep learning" OR intitle:"neural network" OR intitle:"neural networks") |

[1] It is important to note that search terms were adapted for each database to optimize retrieval.

[2] Google scholar was searched by title, serving as a supplementary database, since its alternative full-text search option retrieved a nearly unmanageable number of records (i.e., over 2000).

**Table S2.** The details of included studies

| Study | Data | | | NN | | | DL Training | | | Obstacles |
|---|---|---|---|---|---|---|---|---|---|---|
| | Datasets Accessibility | Validation Data Property | Resolution (mm2 or mm3) | Architectures | Characteristics | Target | Learning Strategy | Training Data Volume (slice) | Input Data Size (voxel) | |
| M. Kidoh et al. [9] | Private | Both | 0.39 × 0.39 | Deep CNN | | Noise | Fully Supervised | 64,800 | 256 × 256 | NR |
| J. Lee et al. [11] | Private | Synthesized | 0.86 × 0.86 | U-Net | | (In-Plane, Rigid) Motion Artifact | Fully Supervised | 425 | 256 × 256 | Hallucination |
| L. Zhang et al. [34] | Private | Synthesized | 1.0 × 1.0 | Vit | Multi-scale Adaptive attention | (In-Plane, Rigid) Motion Artifact | Unsupervised Patch-wise | 13,700 | 16x16 | NR |

| | | | | | | | | | | |
|---|---|---|---|---|---|---|---|---|---|---|
| O. Dabrowski et al. [20] | Private | Both | 1.0 × 1.0 | DenseNet | Frequency-aware | (In-Plane, Rigid) Motion Artifact | Fully Supervised | 1,290 | 256x256 | Hallucination |
| L. Zhang et al. [12] | Private | Synthesized | 1.0 × 1.0 | U-Net | | (In-Plane, Rigid) Motion Artifact | Two-Stage Fully Supervised | 13,700 | 256 × 256 | Hallucination over-smoothing |
| G.G. Potter et al. [13] | Public | Synthesized | 1.0 × 1.0 | U-Net | 3D-aware | (Out-Of-Plane, Elastic) Motion Artifact | Fully Supervised | 331,200 | 256 × 256 | Over-smoothing |
| Y. Zhao et al. [28] | Public | Synthesized | 1.0 × 1.0 | Auto-encoder | Multi-scale Adaptive attention | Ringing Artifact | Fully Supervised | 225,000 | 256 × 256 | Hallucination |
| T. Tajima et al. [27] | Private | In-vivo | 0.3 × 0.3 | Deep CNN | | Noise | Fully Supervised | 39,600 | 768 × 768 | Over-smoothing |
| I. Oksuz [14] | Public | Synthesized | 1.0 × 1.0 | U-Net | | (In-Plane, Rigid) Motion Artifact | Fully Supervised | 16,800 | 128 × 128 | NR |
| B.A., Duffy et al. [15] | Public | Both | 1.15 × 1.15 × 1.1 | U-Net | 3D-aware | (Out-Of-Plane, Rigid) Motion Artifact | Fully Supervised Patch-wise | 518,400 | 128 × 128 × 128 | Over-smoothing |
| S. Liu et al. [16] | Private | Synthesized | 0.8 × 0.8 | U-Net | | Noise | Unpaired Learning | 48,000 | 208 × 256 | Low real-world robustness |
| S. Jung et al. [17] | Private | In-vivo | 0.95 × 0.95 | U-Net | | (In-Plane, Rigid) Motion Artifact | Unpaired Learning | 2,847 | 256 × 256 | Low real-world robustness |
| S. Li et al. [22] | Public | Both | 1.0 × 1.0 | GAN | | Blurring Artifacts | Fully Supervised | 225,000 | 256 × 256 | Low real-world robustness |
| K. Pawar et al. [8] | Public | In-vivo | 0.94 × 0.94 | Resnet | | Noise | Fully Supervised | 157,200 | 256 × 256 | Over-smoothing |
| J. Liu et al. [24] | Private | Synthesized | 0.75 × 1.05 | Resnet | | (In-Plane, Rigid) Motion Artifact | Residual Learning Patch-wise | 125 | 128 × 128 | Over-smoothing |
| S. Li et al. [25] | Public | Synthesized | 1.0 × 1.0 | Resnet | | Rician Noise | Residual Learning Patch-wise | 600 | 41 × 41 | Over-smoothing |
| G. Chen et al. [31] | Public | Synthesized | 0.75 × 0.75 | CycleGAN | | (In-Plane, Rigid) Motion Artifact | Unpaired Learning Patch-wise | Rat brain sMRI images | 128 × 128 | Hallucination Over-smoothing |
| T. Li et al. [32] | Public | Both | 1.0 × 1.0 | CycleGAN | | Intensity Inhomogeneity | Unpaired Learning | NR | NR | Hallucination |
| M.S. Hosseini et al. [36] | Public | In-vivo | 1.0 × 1.0 | PINN | Frequency-aware | Geometric Distortion | Unsupervised | 55,000 | 256 × 256 | Low real-world robustness |
| G. Oh et al. [33] | Public | Synthesized | 0.7 × 0.7 × 0.7 | CycleGAN | Frequency-aware | (In-Plane, Rigid) Motion Artifact | Unpaired Learning | 90,000 | 320×320 | NR |

| | | | | | | | | | | | |
|---|---|---|---|---|---|---|---|---|---|---|---|
| M. Moreno López et al [19]* | The approach proposed in [45] was tested | Public | Synthesized | Not reported (NR) | U-net | Frequency-aware | Gaussian Noise | Unsupervised | NR | 192 × 192 | Over-smoothing |
| | The approach proposed in [46] was tested | Public | Synthesized | NR | U-Net | | Gaussian Noise | Unsupervised | NR | 192 × 192 | Over-smoothing |
| Y. Zhu et al. [29] | | Public | Synthesized | NR | Auto-encoder | | Gaussian Noise | Unsupervised | NR | 256 × 256 | Over-smoothing |
| V. Venkatesh et al. [18] | | Public | Synthetized | NR | U-net | Multi-scale Adaptive Attention | Intensity Inhomogeneity | Fully supervised | 800 | 192 × 192 | Hallucination over-smoothing |
| K. Pawar et al. [26] | | Private | Both | 1.0 × 1.0 × 1.0 | Resnet | | (In-Plane, Rigid) Motion Artifact | Fully supervised | 235, 000 | 256 × 256 | Low real-world robustness |
| Y. Xu et al. [21] | | Public | Synthetized | 0.7 x 0.8 | Residual DenseNet | 3D-aware Adaptive attention | Rician Noise | Fully supervised | 299,400 | 256 × 256 × 5 | Over-smoothing |
| M. Ghaffari et al. [23] | | Public | Synthetized | 0.7× 0.7× 0.7 | GAN | 3D-aware | Blurring & Ringing Artifacts | Fully supervised Patch-wise | 240,000 | 128×128×128 | Hallucination |
| M. Ghahremani et al. [30] | | Public | Synthetized | 1.0 × 1.0 | Auto-encoder | Multi-scale | Rician Noise Intensity Inhomogeneity | Fully supervised | 324,000 | 181×217 | Hallucination |
| M. Safari et al. [37] | | Public | Both | 1.0 × 1.0 × 1.0 | PINN | Adaptive attention | (In-Plane, Rigid) Motion Artifact | Fully supervised | 54,160 | 160 × 192 × 160 | Hallucination Low real-world robustness |
| B. Nghiem et al. [35] | | Public | Both | 1.0 × 1.0 × 1.0 | U-Net | 3D-aware | Ghosting Artifact | Fully supervised | 61,440 | 224 × 218 × 3 | Hallucination |
| A. De Goyeneche Macaya et al. [38] | | Public | Synthetized | 1.0 × 1.0 × 1.0 | PINN | 3D-aware | Blurring Artifact | Fully supervised Patch-wise | NR | 64 ×64 × 64 | Hallucination |
